# Potential Associations Between Psychological Distress and Ambient Air Quality Among Secondary School Teachers in New Jersey

**DOI:** 10.3390/ijerph23030407

**Published:** 2026-03-23

**Authors:** Derek G. Shendell, Juhi Aggarwal, Quincy W. Hunter, Midhat Rehman, Alexa Fiumarelli DeBenedetto, Maryanne L. Campbell

**Affiliations:** 1 New Jersey Safe Schools Program (NJSS), Rutgers School of Public Health (SPH), Rutgers University, 683 Hoes Lane West, 3rd Floor SPH—Suite 399, Office 384/Field Dry Lab 381, Piscataway, NJ 08854, USA; ja880@sph.rutgers.edu (J.A.); qwh1@sph.rutgers.edu (Q.W.H.); mr1638@sph.rutgers.edu (M.R.); mlf159@sph.rutgers.edu (M.L.C.); 2 Department of Environmental & Occupational Health & Justice, Rutgers School of Public Health (SPH), Rutgers University, Piscataway, NJ 08854, USA; af752@scarletmail.rutgers.edu; 3 Rutgers Environmental and Occupational Health Sciences Institute, Piscataway, NJ 08854, USA; 4 Department of Biostatistics and Epidemiology, Rutgers School of Public Health (SPH), Rutgers University, Piscataway, NJ 08854, USA

**Keywords:** Kessler 6+, mental health, outdoor air quality, PM_10_, PM_2.5_, ozone, psychological distress, community-engaged research

## Abstract

**Highlights:**

**Public health relevance—How does this work relate to a public health issue?**

- Both the physical and mental health of the teachers of the world’s children have received renewed attention due to the COVID-19 pandemic plus other societal factors.
- This is an initial environmental epidemiology study with an ecological design to inform future collaborative research on mental health and the effects of ambient/outdoor environmental factors.

**Public health significance—Why is this work of significance to public health?**

- To date, there are few studies of how acute or short-term exposure to ambient or outdoor air pollutants (subject to regulation) potentially affect mental health or psychological distress.
- This study provides recommendations for future research on ambient/outdoor air pollution and the mental health of secondary school teachers, and potentially their adolescent students.

**Public health implications—What are the key implications or messages for practitioners, policy makers and/or researchers in public health?**

- This study’s analyses suggested the estimates of prior 30-day PM_2.5_, as affected by specific events like wildfires and weather factors, showed a significant negative correlation with K6+ scores.
- This study supports the need for more longitudinal research on how particulate matter affects neurological diseases and mental health via indicators or symptoms of psychological distress.

**Abstract:**

Cross-sectional surveys of psychological distress using the Kessler-6 tool (K6+) were conducted among training cohorts per year of New Jersey (NJ) secondary school teachers between January 2022 and December 2024. Data downloaded for 12–18 annual virtual synchronous live session training date ranges related to specified teacher cohorts, consisting of 30 calendar days prior to its date to relate to K6+ questions (575 unique participants across 42 total live sessions). Utilizing data from federal/state air quality monitoring stations (AQMS), we constructed a database of estimated exposures to ambient/outdoor air quality. Cohorts were broken down by school district (SD) and paired with AQMS based on approximate geographic proximity for each SD’s school’s physical address utilizing NJ-GeoWeb. Once addresses were reported and associated with two AQMS, associated reviewed daily criteria pollutant data (2021–2024) were retrieved for particulate matter (PM, PM_10_ and PM_2.5_) and ozone. Data were averaged for relevant stations. Analyses suggested prior 30-day PM_2.5_ showed a significant negative correlation with K6+ scores, −0.32 with PM_2.5_ concentration (*p* = 0.04) and −0.48 with PM_2.5_ AQI (*p* = 0.002); however, wind speed had a positive association, 0.33, with K6+ scores (*p* = 0.03). These results suggested how specific events and meteorological conditions affected ambient air quality for only some of the prior 30 days yet still potentially influenced K6+ scores for some cohorts, e.g., large wildfires then prevailing winds. More research with improved exposure assessment is warranted. This initial environmental epidemiology study with ecological design can inform future collaborative research and practice work on mental health and the effects of environmental factors.

## 1. Introduction

Both the physical and mental health of secondary school teachers have received renewed attention in public health and health policy. To date, some studies have indicated how a working teacher’s actual and perceived health, safety, and wellness—whether positive or negative—influence personal, peer and student wellbeing, academic achievement, emotional wellness, and perceptions of success [1,2,3,4,5,6,7,8,9,10]. Recently, prior studies focused on teachers in New Jersey (NJ), overall [7,8] or among those newer teachers who started their jobs during the COVID-19 pandemic [9,10,11,12]. These prior studies identified the self-care practices that teachers adopted and suggested how teachers could continue to prioritize their physical and mental health. These behaviors included practices about using both cleaning, sanitizing and disinfecting products and personal care products, nutrition choices, sleep habits, exercise routines, and social connections.

Nevertheless, individual behaviors relate far more to the many indoor microenvironments in which we spend time. Teachers spend most of their time indoors, mostly at home or work. This contrasts to time outside in the natural and built environments, which are impacted by water, soil/sediment and air pollutants from both natural and anthropogenic sources of various types/categories and sizes. Outdoor, or ambient, air quality can affect indoor air and environmental quality via mechanical ventilation (even if filtration) and natural ventilation without filtration, especially if inadequate and/or no air cleaners are present to capture smaller size fractions and adsorbed or attached volatile and semi-volatile organic compounds [13,14,15,16,17].

Research has started to identify ambient air pollutants as risk factors for chronic neurological diseases among adults [18,19,20]. This is separate from the well established body of research about specific pollutants and specific cardiovascular and respiratory health outcomes, adverse birth outcomes and cancers of the lung, blood and other vital organs [21,22,23,24,25,26,27]. To date, there are few studies of how acute or short-term exposure to criteria ambient air pollutants (subject in the United States of America (U.S.) to federal and/or state regulation) potentially affect the mental health or psychological distress of adults via visible, documented, or self-reported symptoms of anxiety, depression, emotional stress, or performance on cognitive tests [28,29,30,31,32,33,34].

### 1.1. Background: Current Research Gaps About Mental Health Including Psychological Distress and Outdoor/Ambient Air Pollution Exposure

#### 1.1.1. Inhalable Particulate Matter/Particles (Fine or PM_2.5_, Respirable/Coarse or PM_10_)

Dominski et al. (2021) [23] stated that one of the identified research gaps among the reviewed literature involves psychological and social aspects (mood, stress, and anxiety) and the influence of air pollution and further suggested the need to research and quantify such influence. Dominski et al. further highlighted two 2019 papers—Ali et al. and Braithwaite et al. (further summarized in subsection below) [28,29]—in stating the need to quantify the association between exposure to air pollution and risk of depression, especially regarding particulate matter or particles (PM) in the fine (PM_2.5_) as well as in the respirable or coarse (PM_10_) size fractions. Braithwaite et al. (2019) [29] suggested a possible etiological link between PM exposure and psychological distress, with supporting evidence suggesting associations between long-term PM_2.5_ exposure and depression and possibly anxiety, and short-term PM_10_ exposure and suicide.

When comparing the constituents of inhalable particulates (PM_10_) and fine inhalable particulates (PM_2.5_), PM_2.5_ comprises mutagenic by-products of combustion of fossil fuels, whereas PM_10_ primarily comprises minerals and biological materials [26,27]. While the primary health effects of PM_10_ and PM_2.5_ are well documented, there is limited research on acute and chronic PM_10_ and PM_2.5_ exposure on psychological health.

For example, multiple papers reviewed by Buoli et al. (2018) [30] found a potential association between increased ambient/outdoor PM_2.5_ concentrations and exposure duration and new onset or worsening of depressive symptoms/depression, respectively, and how PM_10_ and O_3_ were among air pollutants associated with an increased risk of self-harm. These authors summarized PM levels associated with exacerbating psychological distress symptoms, increasing emergency department visits, and overall hospitalizations [30].

#### 1.1.2. Ground-Level Ozone (O_3_)

Tropospheric, or ground-level ambient/outdoor ozone (O_3_), is created when volatile organic compounds and nitrogen oxides react with solar radiation [24]. To date, little information exists regarding psychological distress and O_3_. Pinault et al. (2020) [34] identified a positive spatial association between psychological distress and PM_2.5_ and nitrogen dioxide but found less consistent associations between psychological distress and O_3_. Lim et al. (2012) [31] reported a significant association between increased concentrations of O_3_ and symptoms of depression among the elderly, and suggested that O_3_ may impact depression symptoms via inducing oxidative stress. Szyszkowicz et al. (2016) [20] also discussed the potential outcomes associated with induced oxidative stress following O_3_ exposure, including the potential impact it may have on the central nervous system leading to symptoms of depression, and how in previous studies an association between O_3_ and depression and suicide attempts was demonstrated as well as an association between increased levels of O_3_ and increased hospital visits for depression among males and females. These authors also accounted for the impact that seasons have on O_3_ exposure, noting the basic known science of O_3_—concentrations increase during warm months and are lower during cold months [24]—and how associations between suicide and psychological factors may be influenced or confounded by higher ambient temperatures rather than elevated O_3_.

### 1.2. Study Objectives and Goals

To capitalize on the government air quality monitoring stations (AQMS) network in the State of NJ and our separate efforts to assess secondary school teacher mental health during and after the COVID-19 pandemic in 2021–2024 [7,8,11], this initial environmental epidemiology study with an ecological design can inform future collaborative research and practice work on mental health and the effects of environmental factors. Cross-sectional surveys of psychological distress using an updated version of the Kessler-6 tool, the K6+ tool, were conducted among 12 training cohorts of New Jersey (NJ) secondary school teachers from winter of the 2021–2022 school year (SY) to the 2023–2024 SY, i.e., they covered three calendar years, 2022–2024. Our research question was to examine the potential associations between K6+ scores and routinely monitored levels of ambient air pollution and weather variables.

## 2. Materials and Methods

Between 2022 and 2024, NJSS provided 42 work-based learning trainings for a total of 575 NJ CTE teachers (18 cohorts in 2022, 12 cohorts in 2023 and 2024). During these trainings, we assessed symptoms associated with general mental health with Kessler 6 tool (K6+) [35,36,37,38]. For details of use in NJ, see prior studies [8] on the conduct of this non-clinical survey-based outcome assessment of mental health as an indicator of psychological distress in the prior 30 days. The K6+ asks six questions: How often one feels “nervous,” “hopeless,” “restless or fidgety,” “so depressed that nothing could cheer you up,” “that everything was an effort,” and “worthless” on a 5-point Likert scale, with “0” signifying “none of the time” to “4” signifying “all of the time.” The K6+ scores of 0–7 indicate low distress, scores of 8–12 indicate moderate distress, and scores of 13–24 suggest or indicate potential mental distress [35,36,37,38]. However, it must be noted how the K6+ is a tool, and this study neither involved nor conducted formal clinical assessment-to-diagnosis [8,35,36,37,38].

Utilizing data from federal/state air quality monitoring stations (AQMS) [39,40,41], we constructed a database of estimated exposures to ambient/outdoor air quality. For two key criteria air pollutants subject to federal and state regulation in the U.S. and State of NJ, ozone or O_3_ and particulate matter in both the fine or PM_2.5_ and respirable/coarse or PM_10_ size fractions, data were abstracted from up to 20, 17 and three (3) AQMS within the State of NJ, respectively, for each study year 2021–2024. The average/arithmetic mean and minimum–maximum distances between schools (by official physical address) and the primary or 1st assigned AQMS, in miles (kilometers), were approximately 7 (10) and 1–32 (<1–51). For the other or 2nd assigned AQMS, these values in miles (kilometers) were about 10 (16) and 1–37 (<1–60). If two stations were averaged since similar in distance away or because the 1st assigned AQMS had missing data for the date range (cohort’s live session), then it was calculated with equal weighting for this initial environmental epidemiology study with ecological design. In summary, there were only no data for 2021 at Union City High and Paterson (for PM_2.5_) and at Monmouth (for O_3_), and for 2023–2024 at the Newark Firehouse (across these three targeted pollutants). It must also be noted how although data may be reported as being available, this does not indicate completeness of associated data corresponding to specified date ranges for the present study by NJSS. If data were missing/lacking at both the 1st and 2nd assigned AQMS for a specific date range (a cohort’s live session), which was quite limited for this study’s targeted pollutants, then we used the average of the value assigned to teachers in the same cohort’s live session.

Cohorts were broken down by school district (SD) and paired with AQMS based on approximate geographic proximity for each SD’s school utilizing NJ-GeoWeb [42] after retrieving school addresses from NJ School Performance Reports database [43]. Once addresses were reported and associated with 2+ AQMS, associated reviewed daily criteria pollutant data (2021–2024) were retrieved for particulate matter (PM, PM_10_ and PM_2.5_) and ozone. Data downloaded were refined to include those associated with 12 virtual training date ranges related to specified teacher cohorts, including two live session trainings per date range consisting of 30 calendar days prior to its date to relate to K6+ questions. In 2022, there were three live session trainings per date range, to account for the NJ CTE newer teachers initiative, for a total of 42 cohorts in 2022–2024. Data were averaged for relevant stations. No demographic, besides school district and county of work, was collected from the 575 unique participants over the 42 cohorts in this study.

We computed the correlation coefficients between the different aggregate environmental factors: PM_2.5_ and PM_2.5_ AQI averages a month prior to and day of the live session; ozone and ozone AQI averages a month prior to and day of the live session; month prior to temperature (Celsius and Fahrenheit or °C and °F); day of the live session maximum, minimum and average of temperature (°C and °F) and of relative humidity (RH%); day of the live session maximum and average wind speed (in meters per second or m/s); heat index [44]; and humidex and apparent temperature (also see Equation (1) [45] and Equation (2) [46] below) and K6+ score (n = 42). The aggregate K6+ scores were used to calculate Pearson correlation if normally distributed; however, if not normal, then Spearman correlation was used. Normality was determined by the Shapiro–Wilk test. Humidex describes how hot the weather feels to the average person, encapsulating both temperature and humidity. The typical equation to calculate humidex involves dewpoint and temperature; however, we used a variant, which uses RH in % and temperature (in °C) [45]:

(1) $$\mathrm{Humidex}=T+\frac{5}{9}(e-10);\;\mathrm{where}e=6.112\times 10^{\left(7.5T/(237.7+T)\right)}\times \frac{RH}{100}$$

Apparent temperature (AT), which represents an individual’s perceived air temperature, was also calculated via the following equation [46], where AT is in °C, and temperature is T or measured air temperature (°C), wind speed is v (m/s) and RH is in %.

(2) $$\mathrm{AT}=-2.7+1.04T-0.65v+2.0\times \frac{RH}{100}\times 610.78\;\exp\left(\frac{T}{T+238.3}\right)\times 17.27$$

In this study, *p*-values < 0.05 were considered statistically significant. Data were managed using Microsoft Excel and analyzed using SAS analytics software 9.4 (Cary, NC, USA).

## 3. Results

Between the 2021–2022 and 2023–2024 school years, which included calendar years 2022, 2023 and 2024, there were 42 live sessions conducted for a total of 575 participants, who represented each of the 21 counties in the State of New Jersey.

**Figure 1:**
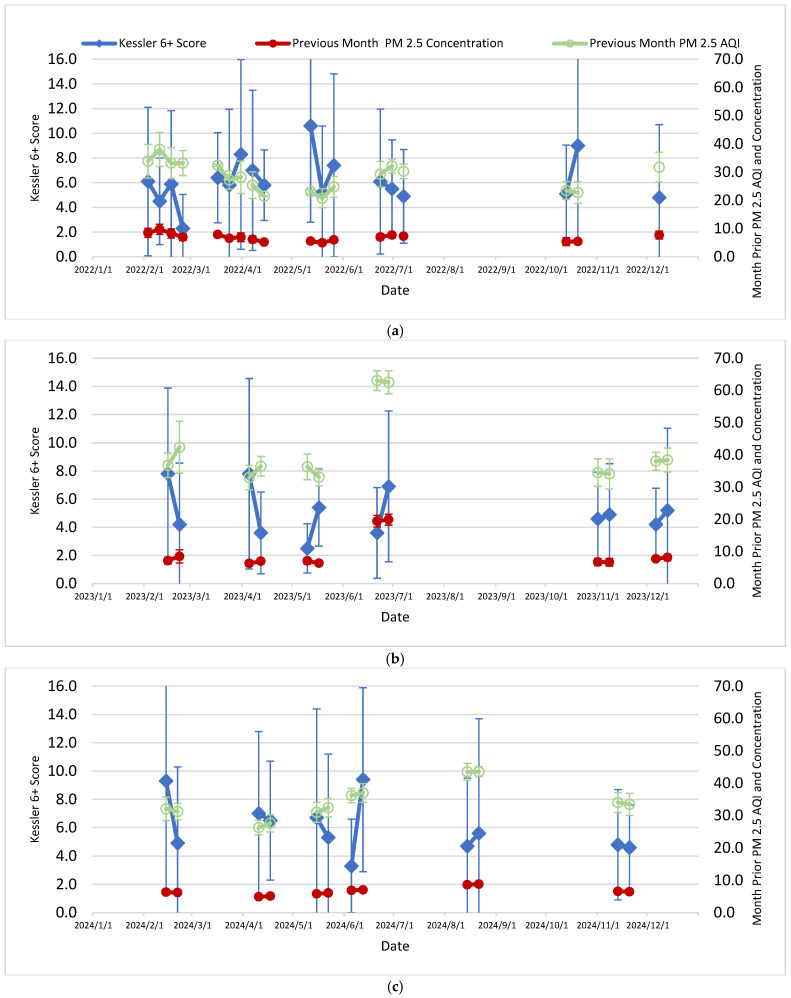
(**a**–**c**): Kessler-6+ score and previous month PM_2.5_ outdoor or ambient air quality index (AQI) and concentration in (**a**) 2022, (**b**) 2023, (**c**) 2024. Please note the error bars may go off scale.

**Figure 2:**
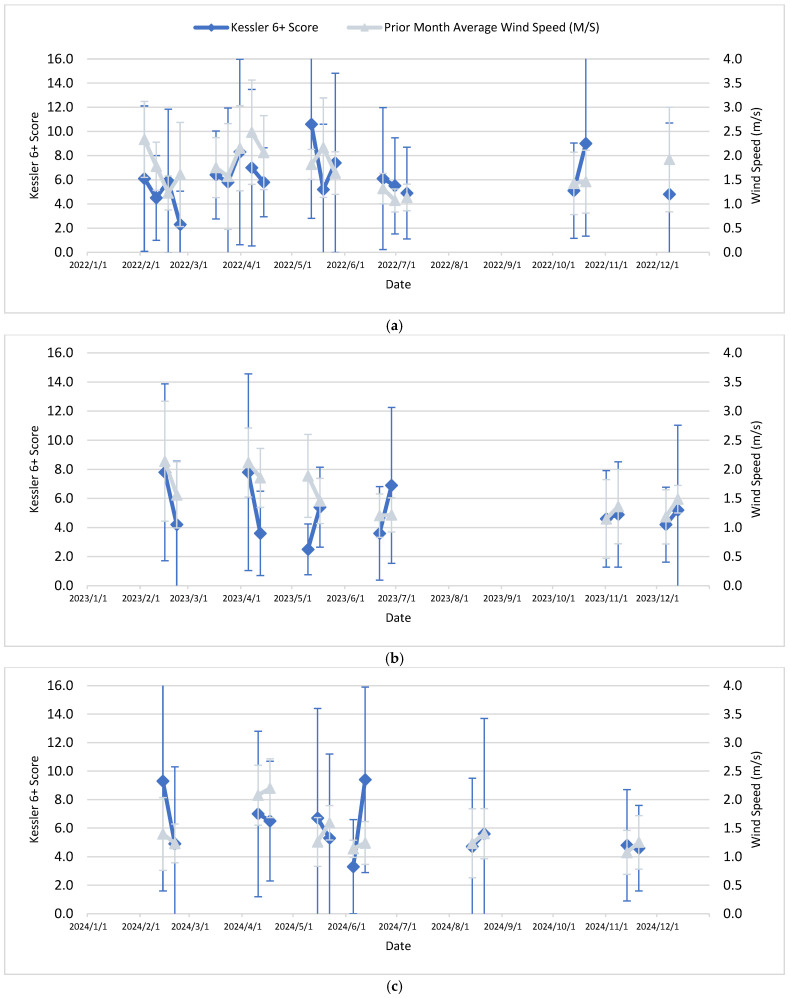
(**a**–**c**): Kessler-6+ score and month prior average wind speed association: (**a**) 2022, (**b**) 2023, (**c**) 2024. Please note the error bars may go off scale.

**Figure 3:**
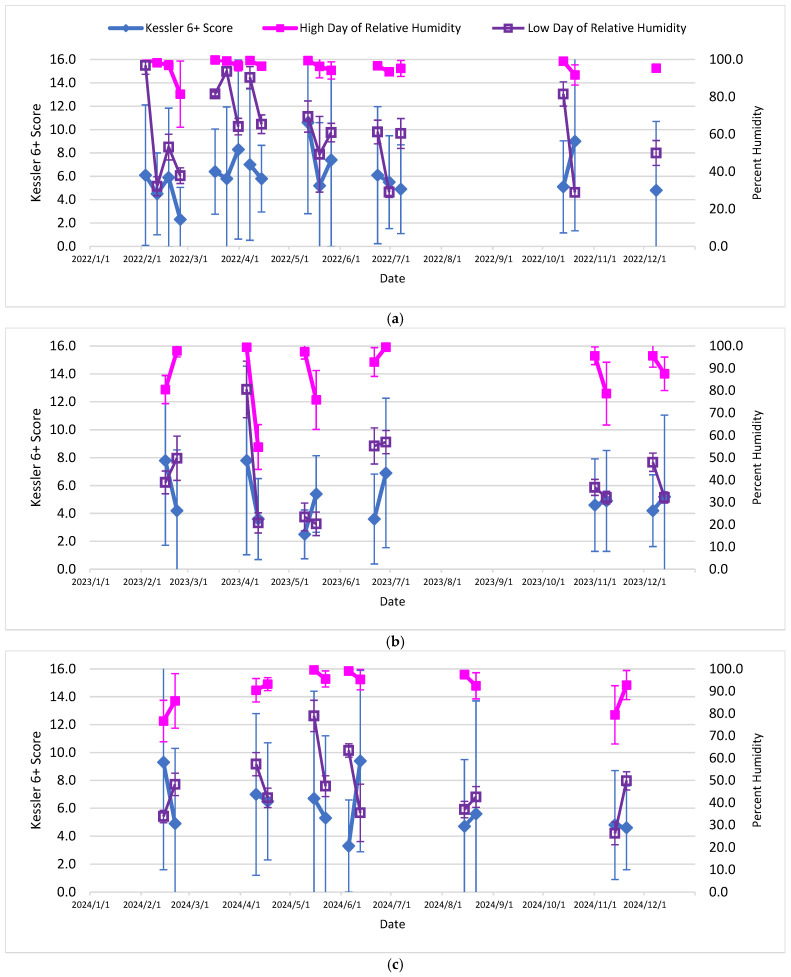
(**a**–**c**): Kessler-6+ score and daily relative humidity association: (**a**) 2022, (**b**) 2023, (**c**) 2024. Please note that maximum relative humidity values > 100% possible per accuracy and precision of sensors. One measurement is above 100% on 6 December 2024. Please note that error bars may go off scale.

Table 1 presents descriptive statistics by calendar year for 2022–2024 for calculated average values or arithmetic means of the study variables. Table 2 then presents, for the same study variables, coefficient and correlation tables and computed statistical associations with computed K6+ scores as an indicator of psychological distress for the 30 calendar days prior to the live session. Finally, Figure 1, Figure 2 and Figure 3, each with parts a–c (by calendar year 2022–2024), present K6+ scores and the previous month’s (30 days) PM_2.5_ AQI, wind speed and daily average relative humidity, respectively, including error bars (calculated as one standard deviation).

The ambient or outdoor environmental factors in this study which correlated with higher K6+ scores overall—an indicator of psychological distress—were PM_2.5_ concentrations and AQI scores the month prior to the live session and the mean of the prior month’s wind speed. This study found a negative association (−0.32) with the increased PM_2.5_ concentration and K6+ scores (*p* = 0.04), and a moderate negative association (−0.48) between PM_2.5_ AQI and K6+ scores (*p* = 0.002). Interestingly, the month prior wind speed had a positive association (0.33) with K6+ scores (*p* = 0.03). The study also found that certain ambient or outdoor environmental factors were only associated with K6+ scores in specific years: PM_2.5_ AQI the month prior to the live session date (K6+ score obtained) was moderately associated in 2022 (*p* = 0.04), and the wind speed was borderline positively associated in 2024 (*p* = 0.07); maximum relative humidity the day of the live session was borderline associated in 2022 (*p* = 0.09) (Figure 3a); however, in 2023 the minimum relative humidity the day of the live session was borderline associated (0.09) (Figure 3b). The study found no associations between psychological distress or K6+ scores and the ozone concentration, ozone AQI, or temperature overall or in the specific study years of 2022–2024.

## 4. Discussion

The average outdoor or ambient air quality index (AQI) for PM_2.5_ between 2022 and 2024 was 33.5, which was lower than the population weighted average in NJ in 2023 at 41.5 [47]. This is notable, given the other weather and climate events recorded in the years of this study [48,49,50,51,52,53,54,55,56,57,58]. Collectively, the data in this study and these events suggest potential acute and chronic impacts on an individual’s mental health, and the K6+ scores provide an indicator of psychological distress.

In early June 2023 (especially 7–8 June 2023), the eastern Canada wildfires affected the eastern U.S. including throughout the State of NJ [48,49,50,51], with a complex mixture of PM_2.5_, PM_10_, inorganic metals and organic compounds in the gas phase and particle phase [51]. This event was part of the prior 30 days relevant to the 4th cohort’s two live session dates (see Figure 1b and Figure 2b regarding late June on the 21st and 28th). Moreover, there were other smaller wildfires in Canada affecting NJ in the months of July, August and October [52]. It must also be noted how December 2023 was the wettest month on record for NJ, including a major precipitation event with high winds on 17–18 December, with rainout and washout of PM_10_, PM_2.5_, ozone and other inorganic and organic chemical pollutants attached to particles [39,40,52]. These events in the fall months of 2023 were relevant to the 5th and 6th cohort’s live session dates (two for each, see Figure 1b, Figure 2b and Figure 3b).

There were also notable weather and climate events within the years of 2022 and 2024. Over 13,000 acres burned in the Pinelands National Forest in southern NJ (in both Atlantic and Burlington counties) in mid-June 2022, and then the dry, warm, windy conditions on 19 June enhanced the Mullica Fire (Wharton State Forest) of 18–21 June, eventually burning over 72,000 acres by the end of the first full day, addressed 20 June [53,54,55,56]. These events were relevant to the 4th cohort’s three live session dates (see Figure 1a and Figure 2a). In addition, July–December 2024 were months of drought relative to historical averages [57,58]. October 2024 was the driest month on record [58]; there were 353 fires responded to throughout the state by the NJ Forest Service in 2024 [57]. November 2024 had record high daily temperatures in NJ [58]. These conditions in general can lead to increased levels of pollutants like PM_10_, PM_2.5_ and O_3_ outdoors. These events and persistent weather conditions were relevant to the 5th and 6th cohort’s live session dates (two for each, see Figure 1c, Figure 2c and Figure 3c).

Signoretta et al. (2019) [19] reported an association between poorer mental wellbeing and perceptions of living in neighborhoods with poor ambient/outdoor air quality, but no significant association between objective measures of ambient/outdoor air pollution like PM_2.5_ and mental wellbeing. The present study was an improvement by using the K6+ tool.

Liu et al.’s 2020 study [32] reported a potential association between mental stress and the indirect influence of self-reported physical symptoms through increasing perceived health effects and attribution to pollution sources, but no direct link between mental stress and real-time PM_2.5_ exposure indoors or outdoors. Furthermore, these authors reported that participant risk perception varied based on indoor vs. outdoor air pollution: the indoor environment was perceived with familiarity; the outdoor environment was attributed with perceived control. The present study did not have real-time measures of PM_2.5_, neither ambient/outdoor nor indoor on the days of each cohort’s live sessions, and thus future research should attempt to assess such exposures for the day of and prior 30 days where people spend most of their time, i.e., at home, work, or school for teachers.

Sass et al. (2017) [18] conducted a study utilizing longitudinal data from the U.S. to further investigate the relationship between air pollution exposure and psychological distress utilizing the Kessler K6+. Moreover, Sass et al. reported how increased ambient/outdoor PM_2.5_ concentrations were positively associated with an increased risk of psychological distress; however, it did not have statistically significant findings for PM_10_ exposure and psychological distress. Their findings, interestingly, were in the opposite direction from this study’s negative statistically significant correlation with PM_2.5_. We believe that our data suggested that certain teacher cohorts were likely differentially impacted by extreme weather events and wildfires affecting ambient air quality for several days within the prior 30 days and thus the average PM_2.5_ value. There is also the clear potential for ecological fallacy, or how the computed group-level ambient PM_2.5_ exposure averages (for each live session within each cohort) may not reflect individual-level associations. Finally, this initial hypothesis-generating study had a limited sample size and was not year-round, i.e., at specific times for training cohorts (575 participants in 42 live sessions across 12 cohorts in each of the three years, 2022–2024). The present study did not have notable findings regarding potential associations or correlations between K6+ scores and the other environmental factors initially targeted, i.e., ambient/outdoor O_3_, T, RH and metrics calculated from these weather variables. It is noted how this may be partly due to fewer cohorts, and thus fewer live sessions and participant teachers being assessed, during months with typically hotter and more humid conditions and higher O_3_ in the mid-Atlantic region (May–September) compared with the late fall–early spring months (October to early December plus late January–April). Pinault et al. (2020) [34] identified a positive spatial association between psychological distress and PM_2.5_ but found less consistent associations between psychological distress and O_3_. Lim et al. (2012) [31] reported a significant association between increased concentrations of O_3_ and symptoms of depression, but specifically among the elderly. These authors also accounted for the impact of seasons on O_3_ exposure; it is well known how concentrations of O_3_ increase during warm months and are lower during cold months [40].

### Study Limitations

The estimated exposure database portion of this project highlights the current limitations with AQMS data collection. It was observed how U.S. EPA and NJ DEP AQMS are only typically located in densely populated areas, mostly urban and suburban areas plus some rural areas like national parks or recreation areas for background sites, and are typically close in proximity. Additionally, not every AQMS collected the same data, and not every AQMS collected data during date ranges corresponding to NJSS K6+ implementation (see Materials and Methods section above). Thus, there were gaps in the exposure database due to limited data availability for this initial environmental epidemiology study with an ecological design; however, this study does inform hypotheses or questions and directions for future research. Furthermore, we did not do distance-weighting, only averaging, when using ambient/outdoor air quality data nearest to the participant’s school from both the primary/first assigned and other/second assigned AQMS.

There were also known limitations to the outcome’s measurement data for this project’s K6+ scores correlation analyses. Even with 575 volunteer teachers in NJ CTE across 42 live sessions for three years of teacher cohorts, this is a relatively small sample size for effective correlation analyses. In addition, there may have been potential selection bias introduced. These NJ CTE participant secondary school teachers are completing required courses to gain knowledge and skills to supervise students and other staff members, i.e., may have different levels of psychological distress versus other K-12 teachers. The K6+ scores were also self-reported, which may have led to biases such as social desirability or self-servicing bias.

Finally, it is noted how this initial environmental epidemiology study with an ecological design did not have any formal statistical corrections to account for regarding potential multiple comparison Type I errors within the correlation analyses of the participant K6+ scores and the environmental variables. Additionally, individual-level demographics, personal health information, and identifiers cannot be included in the dataset for statistical analysis per IRB approval, i.e., to maintain the confidentiality of individuals as well as NJ districts/schools. Lastly, there is the known issue with this study design of ecological fallacy with respect to the use of geographical area estimations of ambient/outdoor air pollution exposure. Future research with more robust cohort and/or repeated cross-sectional measurement designs across various seasons would enhance exposure assessment, control for inter- and intra-seasonal differences, and do multiple comparisons statistical procedures.

## 5. Conclusions

This study’s initial analyses of the 2021–2024 school year and calendar year data suggested prior 30-day estimated that exposure to outdoor or ambient air concentrations of fine particulate matter or PM_2.5_ was negatively correlated to adult secondary school teacher Kessler/K6+ scores, an indicator of psychological distress, throughout New Jersey. These results, which were opposite in direction from the limited prior research, can encourage discussion and help inform future collaborative research among teams of public health, atmospheric science and environmental science researchers interested in the physical and mental health of K-12 school teachers. Future studies with more rigorous epidemiological designs and geographically precise measures are warranted to assess the potential effects of exposures to outdoor or ambient air pollutants and other environmental factors on mental health year-round, across seasons/times of year. The results of this study and future studies then provide evidence to guide policy reforms in urban planning, zoning regulations, emission standards, clean energy adoption, transportation systems, etc.

## Figures and Tables

**Table 1:** Averages or arithmetic means of study variables.

Variable	2022 Mean (n = 18)	2023 Mean (n = 12)	2024 Mean (n = 12)	Overall Mean (n = 42)
PM_2.5_ average month prior to live session (micrograms per cubic meter of air µg m^−3^)	6.87	9.21	6.59	7.46
PM_2.5_ AQI average month prior to live session	28.41	40.73	34.03	33.54
PM_2.5_ average day of live session (µg m^−3^)	7.25	6.92	6.50	6.94
PM_2.5_ AQI average day of live session	30.00	34.85	34.45	32.66
Ozone average month prior to live session (parts per million or ppm)	0.04	0.04	0.04	0.04
Ozone AQI average month prior to live session	34.37	35.75	38.16	35.85
Ozone average day of live session (ppm)	0.03	0.04	0.04	0.04
Ozone AQI average day of live session	33.21	38.06	37.12	35.71
Month prior temperature (Celsius)	9.38	10.40	13.47	10.84
Month prior relative humidity (%)	66.75	69.16	71.37	68.76
Month prior wind speed (meters per second or m/s)	1.72	1.55	1.43	1.59
Daily relative humidity high (%)	96.11	88.00	91.41	92.45
Daily relative humidity low (%)	61.46	41.26	46.85	51.51
Daily relative humidity average (%)	79.28	64.94	70.99	72.81
Daily temperature high (Celsius, °C)	16.69	16.54	18.54	17.17
Daily temperature low (°C)	7.20	6.02	8.40	7.21
Daily temperature average (°C)	12.25	11.39	13.25	12.29
Max wind speed (m/s)	8.74	8.55	6.52	8.05
Average wind speed (m/s)	1.67	1.67	1.48	1.61
Humidex	13.39	11.01	14.70	13.08
Heat index	52.89	50.51	54.48	52.66
Apparent temperature	6.96	5.90	9.69	7.44
Kessler or K6+ score	6.15	5.06	6.01	5.80

**Table 2:** Coefficient and correlation tables for study variables and statistical association with computed Kessler 6+ scores.

Year	2022		2023		2024		Overall	
Variable	Coefficient	*p*-Value	Coefficient	*p*-Value	Coefficient	*p*-Value	Coefficient	*p*-Value
PM_2.5_ average month prior to live session (micrograms per cubic meter of air, µg m^−3^)	−0.36	0.15	−0.23	0.46 ^a^	−0.23	0.46	−0.32	0.04 **^a^
PM_2.5_ AQI average month prior to live session	−0.48	0.04 **	−0.27	0.39 ^a^	−0.24	0.45	−0.47	0.002 ***^a^
PM_2.5_ average day of live session (µg m^−3^)	0.13	0.61	0.33	0.29	−0.33	0.30	0.13	0.38
PM_2.5_ AQI average day of live session	0.13	0.62	−0.15	0.64	−0.37	0.24	−0.14	0.38
Ozone average month prior to live session (parts per million or ppm)	0.23	0.37	−0.17	0.6	0.06	0.86 ^a^	0.01	0.96 ^a^
Ozone AQI average month prior to live session	0.23	0.36	0.27	0.39	−0.20	0.53	0.15	0.36
Ozone average day of live session (ppm)	0.01	0.98	0.03	0.93	0.23	0.48	0.03	0.87
Ozone AQI average day of live session	0.06	0.80 ^a^	0.01	0.96 ^a^	0.20	0.54	0.18	0.26 ^a^
Month prior temperature (Celsius, °C)	0.20	0.44	−0.23	0.48	−0.28	0.39	−0.03	0.86
Month prior relative humidity (%)	−0.25	0.31	−0.11	0.73	−0.01	0.78	−0.16	0.30
Month prior wind speed (meters per second or m/s)	0.18	0.48	0.33	0.29	0.55	0.07 *^a^	0.33	0.03 **^a^
Daily relative humidity high (%)	0.41	0.09 *^a^	0.11	0.74 ^a^	0.29	0.36	0.15	0.34 ^a^
Daily relative humidity low (%)	0.20	0.44	0.51	0.09 *	−0.21	0.51	0.25	0.11
Daily relative humidity average	0.32	0.19	0.35	0.26	−0.33	0.29	0.24	0.13
Daily temperature high (°C)	0.30	0.22	0.08	0.80	−0.23	0.47	0.06	0.71
Daily temperature low (°C)	0.18	0.48	0.08	0.80	−0.24	0.44 ^a^	0.07	0.67
Daily temperature average (°C)	0.24	0.33	0.06	0.86	−0.18	0.58	0.06	0.72
Max wind speed (m/s)	0.00	0.99	0.42	0.17	0.42	0.18	0.13	0.40
Average wind speed (m/s)	0.08	0.76 ^a^	0.26	0.41	0.39	0.21 ^a^	0.19	0.24 ^a^
Humidex	0.24	0.34	0.13	0.68	−1.7	0.6	0.08	0.63
Heat index	0.26	0.30	0.08	0.81	−0.18	0.57	0.07	0.66
Apparent temperature	0.22	0.38	0.02	0.96	−0.24	0.46	0.03	0.87

* Correlation significance *p* < 0.1, ** correlation significance *p* < 0.05, *** correlation significance *p* < 0.01. ^a^ Not normal (*p* < 0.05); Spearman correlation used.

## Data Availability

The original contributions presented in this study are included in the article. Further inquiries can be directed to the corresponding author.

## Abbreviations

The following abbreviations are used in this manuscript:

AQMS	Air Quality Monitoring Station
CTE	Career and Technical Education
K6+	Kessler 6+ Tool (for score as indicator of psychological distress during the prior 30 days)
NJDEP	New Jersey Department of Environmental Protection
NJSS	New Jersey Safe Schools Program
O_3_	Ozone
PM	Particulate Matter (PM_2.5_ is fine fraction, PM_10_ is respirable or coarse fraction)
SY	School Year
USEPA	United States Environmental Protection Agency
WBL	Work-Based Learning
